# Data documenting the comparison between the theoretically expected values of free sugars mass isotopomer composition with standards using GC–MS and LC-HRMS for Metabolic Flux Analysis

**DOI:** 10.1016/j.dib.2017.03.038

**Published:** 2017-03-31

**Authors:** Sébastien Acket, Anthony Degournay, Franck Merlier, Brigitte Thomasset

**Affiliations:** Sorbonne Universités, Génie Enzymatique et Cellulaire, FRE CNRS 3580, Université de Technologie de Compiègne, 60203 Compiègne Cedex, France

**Keywords:** Metabolic flux analysis, Isotopic enrichments of free sugars, Mass spectrometry, LC-HRMS

## Abstract

The data presented in this article are related to the research article entitled “13C labeling analysis of sugars by high resolution-mass spectrometry for Metabolic Flux Analysis” (Acket et al., 2017) [1]. This article provides data concerning the comparison between the theoretically expected values of free sugars mass isotopomer composition with standards using our previous methods using low resolution mass spectrometry by GC–MS (Koubaa et al., 2012, 2014) [2,3], and your new method using high resolution-mass spectrometry (LC-HRMS) for Metabolic Flux Analysis [1]. For discussion and a more comprehensive data interpretation and analysis, please refer to Acket et al. (2017) [1].

**Specifications Table**

**Table:** 

Subject area	Biology, Analytical Chemistry
More specific subject area	Metabolic Flux analysis, mass spectrometry, isotopomers, free sugars
Type of data	Figure and Table
How data was acquired	UPLC instrument Agilent 1290 Infinity coupled with high resolution–mass spectrometry (HR-MS Q-TOF UHD 6538) from Agilent Technologies.
	Compounds were separated on a Thermo Hypersil Gold Hilic column (2.1×150 mm, 3 µm).
Data format	Analyzed Raw
Experimental factors	These are described in the text description of the data
Experimental features	These are described in the text description of the data
Data source location	Laboratory Génie Enzymatique et Cellulaire, FRE CNRS 3580, Sorbonne Universités, Université de Technologie de Compiègne, 60205 Compiègne, France
Data accessibility	Data with article

**Value of the data**

• The data show the comparison of mass spectrum of free sugars standards to quantify the isotopic enrichments using our previous method (GC–MS using low resolution mass spectrometry) [2], [3], and our new method (LC-HRMS) [1].
• The isotopic enrichment quantification of the free sugars with the new method [1] is carried out without derivatization (with a saving of time in the sample preparation) and directly on the molecular ion (M–H^+^) (easy to quantify the isotopic enrichments of all the carbons of the molecule), unlike our previous method [2], [3].
• Data show a better accuracy in mass isotopomer analysis compared to our previous method [2], [3] due to the contribution of high resolution mass spectrometry.
• These data are useful for determining the isotopic enrichments of free sugars for bacteria, yeasts, animals and plants cells, for isotopic profiling or for Metabolic Flux Analysis.

## 1 Data

**Fig. 1:**
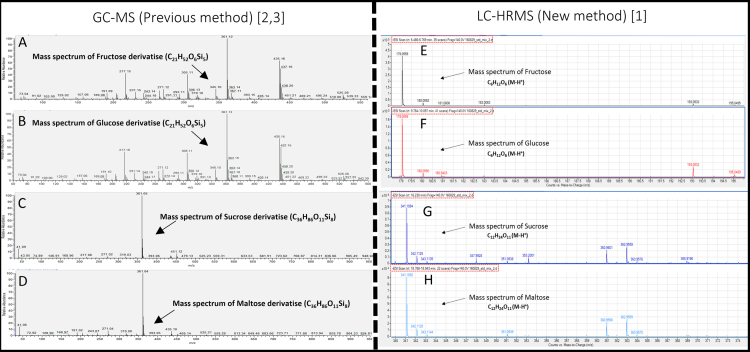
Comparison of mass spectrum of the free sugars used to quantify the isotopic enrichments between our previous method (GC–MS) [2], [3] and our new method (LC-HRMS) [1]. GC–MS mass spectrum derivatise of fructose (A), glucose (B), sucrose (C), maltose (D); LC-HRMS mass spectrum of fructose (E), glucose (F), sucrose (G), maltose (H).

The data being shared (Fig. 1) consists in a comparison of mass spectrum and chromatogram of the free sugars used to quantify the isotopic enrichments between our previous method using low resolution mass spectrometry (GC–MS), [2], [3] and our new method using high-resolution mass spectrometry (LC–MS) [1]. Chromatograms showing separation of free sugars are available, in Ref. [2], [3] for GC–MS (previous method), in Ref. [1] for LC-HRMS (new method).

The accuracy of the isotopic mass values with the theoretical expected values in LC-HRMS compared with our previous method in GC–MS [2], [3] is presented in Table 1.

## 2 Experimental design, materials and methods

### 2.1 Chemicals and standard preparation

All of the free sugars standards were purchased from Sigma-Aldrich Co (St. Louis, MO, USA). For LC-HRMS experiment, a mix of 100 µg ml^−1^ of free sugars was prepared and injected into LC-HRMS as described in Ref. [1]. For GC–MS analysis, the derivatization agent was BSTFA [2], [3]. Briefly, free sugars standards (40 mg) were dissolved in 400 μl of *N*,*N*-dimethylformamide (Sigma–Aldrich) containing 0.1% pyridine (Fluka), and then 50 μl of *N*,*O*-bis(trimethylsilyl)trifluoroacetamide (BSTFA kit from Sigma–Aldrich) was added. The mixture was heated for 30 min at 80 °C to obtain Si(CH_3_)_3_ derivatives of the contained sugars.

### 2.2 LC-HRMS conditions

Each standards and standards mixture was run in triplicates on a HPLC (UPLC 1290 Infinity) coupled with high resolution-mass spectrometry (HR-MS Q-TOF UHD 6538) from Agilent Technologies (Agilent Technologies Inc., GA, USA) as described in ref [1]. Briefly, 5 µL standard was injected using an autosampler (Agilent 1300 series G1329-90010) onto a Thermo Hypersil Gold Hilic column (2.1×150 mm, 3 µm) at 20 °C. Free sugars were chromatographically separated by an increasing ammonium acetate gradient, at a flow rate of 0.5 ml/min. The mobile phase gradient selected was 5 mM ammonium acetate in water (A) and 100% acetonitrile (B): starting from 3% A for 4 min followed by a linear increase to 80% A until 30 min. Cleaning of the column was achieved with 90% B for 5 min. The HRMS analysis was performed with a hybrid quadrupole/Tof mass spectrometer (Q-TOF UHD 6538) from Agilent Technologies. The mass spectra were acquired using a dual electrospray ionization in negative-ion mode. The source temperature was set up at 200 °C. The nebulization gas, the ion spray voltage and the fragmentor were adjusted to 30 psi, 3.5 kV and 140 V respectively. The range of mass detected on time of flight was 50 *m*/*z* and 1050 *m*/*z*, with a scan of two spectra per second.

### 2.3 GC–MS conditions

The free sugars derivatises were analysed using the method presented in Ref [2], [3]. In brief, analysis were performed using a ThermoFisher TSQ Quantum GC triple quadrupole GC–MS device with an equity-5 capillary GC column (5% phenyl-methyl-siloxane diphenylpolysiloxane, 30 m×0.25 mm) from Sigma–Aldrich). The GC conditions were as follows. The GC oven temperature was first set to 120 °C and held for 5 min, then increased to 270 °C at 4 °C/min, and finally increased to 320 °C at 20 °C/min. The injection temperature was fixed to 300 °C, and the injection mode was set to split with a split ratio of 5. The separation of carbohydrates was performed onto this column, under a helium carrier gas flow set up at 1.5 ml/min. For the MS, ion source and the interface temperatures were set to 320 °C with chemical ionization (CI) with CH_4_ at 2 ml/min in positive mode. Detection was in full scan mode between *m*/*z* 30 and 950 with an event time of 0.2 s.

### 2.4 Data processing

For LC-HRMS, data were acquired and processed using MassHunter B.07 software, whereas for GC–MS, data were acquired and processed using Xcalibur software.

## Figures and Tables

**Table 1:** Comparison of the theoretically expected values of mass isotopomer composition between the mass isotopomer composition from standard molecule and with the experimental mass isotopomer composition obtained in our previous method in GC–MS [2], [3].

	GC–MS (Previous methods) [2], [3]^a^					LC-HRMS (New method) [1]^b^			
	Fragment	Theoritical mass^c^	Standard	Error^d^		[M–H]^−^	Theoritical mass^c^	Standard	Error^d^
Glucose derivatise C_21_H_52_O_6_Si_5_					Glucose C_6_H_12_O_6_				
*m*0	525.2	100.00	100.00	0.00	*m*0	179.0561	100.00	100.00	0.00
*m*+1	526.2	47.84	54.72	6.88	*m*+1	180.0595	6.84	5.95	0.89
*m*+2	527.2	28.68	32.08	3.40	*m*+2	181.0607	1.43	1.31	0.12
*m*+3	528.2	9.24	8.15	1.09	*m*+3	182.0639	0.09	0.11	0.02
*m*+4	529.2	3.03	2.58	0.45	*m*+4	183.0654	0.01	0.00	0.01
*m*+5	530.2	0.72	0.12	0.61	*m*+5	184.0683	0.00	0.00	0.00
*m*+6	531.2	0.17	0.02	0.15	*m*+6	185.0750	0.00	0.00	0.00
Fructose derivatise C_21_H_52_O_6_Si_5_					Fructose C_6_H_12_O_6_				
*m*0	525.2	100.00	100.00	0.00	*m*0	179.0561	100.00	100.00	0.00
*m*+1	526.2	47.84	52.21	4.37	*m*+1	180.0595	6.84	5.98	0.86
*m*+2	527.2	28.68	30.09	1.41	*m*+2	181.0607	1.43	1.28	0.15
*m*+3	528.2	9.24	7.08	2.16	*m*+3	182.0639	0.09	0.06	0.03
*m*+4	529.2	3.03	2.21	0.83	*m*+4	183.0654	0.01	0.00	0.01
*m*+5	530.2	0.72	0.35	0.37	*m*+5	184.0683	0.00	0.00	0.00
*m*+6	531.2	0.17	0.14	0.03	*m*+6	185.0750	0.00	0.00	0.00
Sucrose derivatise C_36_H_86_O_11_Si_8_					Sucrose C_12_H_24_O_11_				
*m*0	361.2	100.00	100.00	0.00	*m*0	341.1089	100.00	100.00	0.00
*m*+1	362.2	32.00	29.90	2.10	*m*+1	342.1124	13.64	11.98	1.66
*m*+2	363.2	15.52	16.04	0.52	*m*+2	343.1139	3.12	2.84	0.28
*m*+3	364.2	3.37	3.09	0.28	*m*+3	344.1169	0.34	0.24	0.10
*m*+4	365.2	0.82	0.89	0.07	*m*+4	345.1187	0.04	0.03	0.01
*m*+5	366.2	0.12	0.04	0.08	*m*+5	346.1214	0.00	0.00	0.00
*m*+6	367.2	0.02	0.21	0.20	*m*+6	347.1297	0.00	0.00	0.00
*m*+7	368.2	0.00	0.00	0.00	*m*+7	348.1327	0.00	0.00	0.00
*m*+8	369.2	0.00	0.00	0.00	*m*+8	349.1359	0.00	0.00	0.00
*m*+9	370.2	0.00	0.00	0.00	*m*+9	350.1398	0.00	0.00	0.00
*m*+10	371.2	0.00	0.00	0.00	*m*+10	351.1414	0.00	0.00	0.00
*m*+11	372.2	0.00	0.00	0.00	*m*+11	352.1466	0.00	0.00	0.00
*m*+12	373.2	0.00	0.00	0.00	*m*+12	353.1490	0.00	0.00	0.00
Maltose derivatise C_36_H_86_O_11_Si_8_					Maltose C_12_H_24_O_11_				
*m*0	361.2	100.00	100.00	0.00	*m*0	341.1089	100.00	100.00	0.00
*m*+1	362.2	32.00	29.44	2.56	*m*+1	342.1124	13.64	11.96	1.68
*m*+2	363.2	15.52	16.15	0.63	*m*+2	343.1139	3.12	2.82	0.30
*m*+3	364.2	3.37	3.53	0.16	*m*+3	344.1169	0.34	0.22	0.12
*m*+4	365.2	0.82	1.07	0.25	*m*+4	345.1187	0.04	0.02	0.02
*m*+5	366.2	0.12	0.24	0.12	*m*+5	346.1214	0.00	0.00	0.00
*m*+6	367.2	0.02	0.08	0.07	*m*+6	347.1297	0.00	0.00	0.00
*m*+7	368.2	0.00	0.00	0.00	*m*+7	348.1327	0.00	0.00	0.00
*m*+8	369.2	0.00	0.00	0.00	*m*+8	349.1359	0.00	0.00	0.00
*m*+9	370.2	0.00	0.00	0.00	*m*+9	350.1398	0.00	0.00	0.00
*m*+10	371.2	0.00	0.00	0.00	*m*+10	351.1414	0.00	0.00	0.00
*m*+11	372.2	0.00	0.00	0.00	*m*+11	352.1466	0.00	0.00	0.00
*m*+12	373.2	0.00	0.00	0.00	*m*+12	353.1490	0.00	0.00	0.00

a Method based on the standard trimethylsilyl derivatives of saccharides for isotopomer analysis in GC–MS using chemical ionization as described in [2,3]. For isotopomer analysis of glucose and fructose, the major peak in the CI spectrum containing the whole carbon skeleton is 525.2 (loss of CH3) were used. The dominated by peak 361.2 (containing the whole carbon skeleton) of maltose and sucrose were used for isotopomer analysis.

b Method without derivatives procedures. For isotopomer analysis, the peak containing the whole carbon skeleton is 179.0561 [M–H]^−^ for glucose and fructose and 341.1089 [M–H]- maltose and sucrose were used.

c The theoretical mass was obtained using the software Isotope Distribution Calculator from MassHunter software Agilent Technologies.

d Errors correspond to the absolute value of the difference between the theoretical value and the experimental value.
