# Supplementary material for: Data documenting the comparison between the theoretically expected values of free sugars mass isotopomer composition with standards using GC–MS and LC-HRMS for Metabolic Flux Analysis
Source: Data Brief. 2017 Mar 31;12:108–12. doi: 10.1016/j.dib.2017.03.038 (PMC5384292; doi:10.1016/j.dib.2017.03.038)
Supplement: Supplementary file 1 — Supplementary material [file mmc1.docx]

**CONFLICT OF INTEREST FORM**

**Title of the paper:** Data documenting the comparison between the theoretically expected values of free sugars mass isotopomer composition with standards using GC-MS and LC-HRMS for Metabolic Flux Analysis

**Authors:** Sébastien Acket, Anthony Degournay, Franck Merlier, Brigitte Thomasset

Behalf of all authors, the corresponding author of this paper declare no conflicts of interest associated with this publication and there has been no significant financial support for this work that could have influenced its outcome.

Corresponding author

Dr. Sébastien Acket

Sorbonne Universités
Université de Technologie de Compiègne
FRE CNRS 3580 - Génie Enzymatique et Cellulaire

Rue du Dr Schweitzer

CS 60319, 60203 Compiègne Cedex France

sebastien.acket@utc.fr
